# Gender gap in surgical societies awards

**DOI:** 10.1007/s00464-024-11440-3

**Published:** 2025-01-10

**Authors:** Karimatu Jalloh, Amanda Bader, Leslie M. Okorji, Rachel Kelz, Talar Tatarian, Maria S. Altieri

**Affiliations:** 1 Department of Surgery, University of Pennsylvania, 800 Walnut St 20th Floor, Philadelphia, PA 19107 USA; 2 Thomas Jefferson Hospital, University of Pennsylvania, 3400 Civic Center Blvd 3rd floor, Philadelphia, PA 19104 USA; 3 Thomas Jefferson University, 1100 Walnut St 5th Floor, Philadelphia, PA 19107 USA

**Keywords:** Gender gap, Gender disparities, Surgical societies, Surgical awards

## Abstract

**Introduction:**

Despite remarkable progress, gender inequality in medicine remains a significant issue. This disparity extends beyond clinical practices and educational programs; it is also evident in the recognition and awards received by surgeons. Underrepresentation of women in Surgical Society awards is a multi-layered issue that needs a holistic approach since these awards are used to hire, promote, and advance surgeons.

**Methods and Procedures:**

A retrospective observational study was performed between 1936 and 2023 on all recipients of awards from 22 surgical societies. The study examines the relationship between recipient gender and award year. Medians, and interquartile ranges (IQRs) were used for continuous data, and frequencies and percentages were used for categorical data. Chi-square and Wilcoxon rank sum tests were used to compare female and male recipients. Multiple logistic regression was used to estimate the probability that a female will receive an award.

**Results:**

A total of 2588 awards were given out between 1936 and 2023. Among the 2588 awards, 2024 went to male surgeons, and 564 to female surgeons. Since 1936, there have been 0–25 women awarded annually, with a proportion of female awardees between 0 and 0.5. Since 1936, the proportion of women awardees has increased significantly (*p* < 0.01). Since 2006, female award winners have increased by 0.7% (95% CI 0.007–0.008, *p* = 0.001) when controlling for surgical societies. A woman’s odds of receiving an award from a surgical society have increased by only 3% per year since 2006 (OR 1.03, 95% CI 1.01–1.07, *p* = 0.004). Accordingly, female surgeon awards grew from 0.22 in 2006 to 0.35 in 2023.

**Conclusion:**

Female surgeons’ continuous underrepresentation in Surgical Society awards is a crucial issue. The selection process of surgical societies needs to be more intentional as female recipients have steadily increased in recent years. Closing this gender gap is not only a matter of fairness but also imperative for the field’s progress.

Despite the significant increase in female representation among medical students, the AAMC reports that women continue to make gains in 2022–23, making up 57% of the applicants, 56% of matriculants, and 54% of the total enrolled [1, 2]. Despite the increase in the number of female medical students, women in leadership positions or receiving recognition remains disproportionately low compared to their male counterparts. Institutions continue to rely on the pipeline theory, which suggests that women will eventually achieve the same level of success in medicine as men. However, this theory serves as a passive mechanism to maintain the status quo rather than actively addressing gender disparities [3]. Introduced by Walter R. Mahler in the 1970s, the pipeline theory outlines the progression of individuals through organizational stages, implying that increased representation of women in entry-level positions will lead to greater female presence in top leadership roles. Despite its widespread adoption, research indicates that this theory has not resulted in equitable outcomes.

Contrary to the assumptions of the pipeline theory, studies highlight persistent barriers hindering women’s advancement in medicine. While academic institutions often cite the need for more qualified women in leadership positions, data reveals that women are not absent from the pipeline’s start but are disproportionately excluded from top-tier roles. Studies illustrate a stark underrepresentation of women, especially those from underrepresented minority groups, in leadership positions within academia. Women comprise only 33% of the tenure-track faculty, occupy 39% of dean positions at top American research institutions of higher education, 38% of provosts, 30% of presidencies, and 37% of seats on public institutional boards [1, 4, 5] Despite women outnumbering men in earning bachelor’s, master’s, and doctoral degrees, their representation diminishes as they ascend the career ladder.

Surgical society awards are highly influential in the careers of surgeons, particularly for female surgeons, as they serve as an important measure of success and recognition within the medical community. These awards not only acknowledge past accomplishments but also provide valuable support for future endeavors, which can greatly enhance an individual’s professional standing and advancement [1].

Notably, these awards play a vital role in advancing equality, promotions, recognition, diversity of perspectives, representation, meritocracy, and academic progress, all of which are areas where female surgeons often face obstacles [1, 2]. To tackle the gender imbalance in the distribution of these awards across surgical societies, it is necessary to critically examine the award criteria, the composition of decision-making committees, and the societal and institutional biases that may impact the selection process. This study aims to examine the gender of society award recipients across major American surgical societies during the past 86 years.

## Methods

This was a retrospective observational study. Award recipient information was collected from twenty-two surgical society websites or associated foundations. Awards granted between the years 1936 and 2023 were included in the analysis, Awards that were granted to individuals who were not physicians were excluded.

Relevant data such as the award description, the recipient’s name, gender, and the year of the recipient were collected for the purposes of this study. The gender of the award recipients was determined by conducting internet searches to find photographs or gender-specific pronouns associated with each individual’s name. The data collection process, gender determination, and award categorization for each society were carried out by one investigator and independently verified by a second and a third investigator.

Awards given to trainees (residents or fellows) were categorized separately from awards not specifically intended for trainees. The awards were further classified based on their descriptions into three categories: “achievement,” which acknowledged an individual’s overall contributions to their field, “public service,” which recognized humanitarian or volunteer efforts, and “research,” which highlighted scientific advancements or provided grants for future research. In cases where multiple authors were associated with a presentation or publication, the first author was considered the award recipient.

The primary exposure of this study was award recipient gender, and the primary outcome measure was the number of awards received by each gender. A secondary outcome was to assess the changes in patient of award recipient gender over time.

Descriptive statistics were used to calculate medians and IQRs for continuous data and frequencies/percentages for categorical data. Statistical comparisons between female and male award recipients were made using Wilcoxon rank sum and chi-square tests, respectively. A multivariate logistic regression was used to assess variables associated with award receipt to a woman in any given year.

## Results

**Fig. 1:**
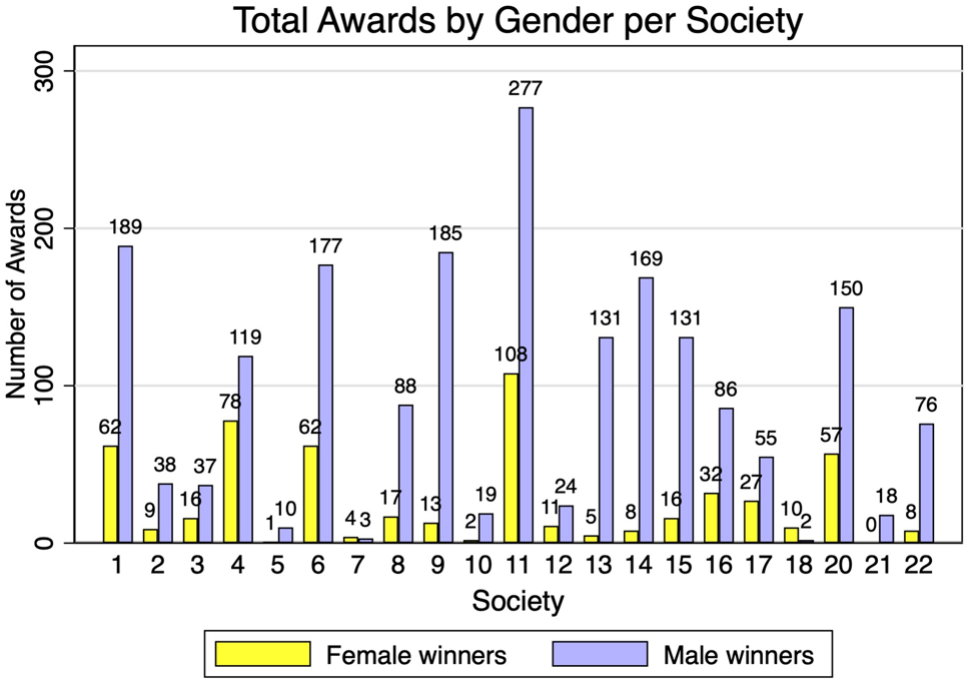
Total awards by gender per society

**Fig. 2:**
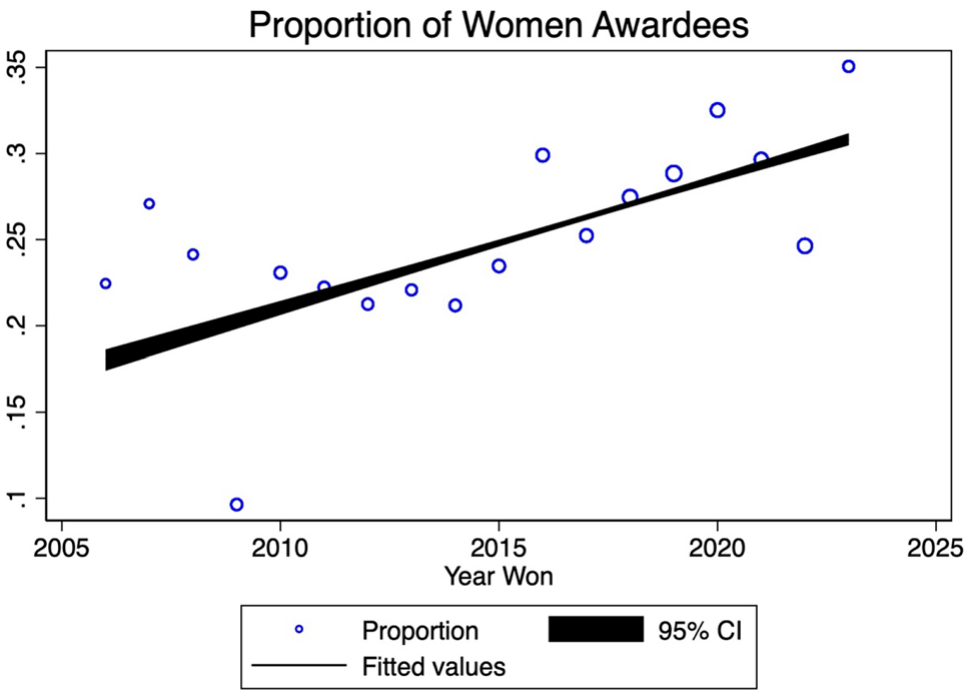
Proportion of women awardees

**Fig. 3:**
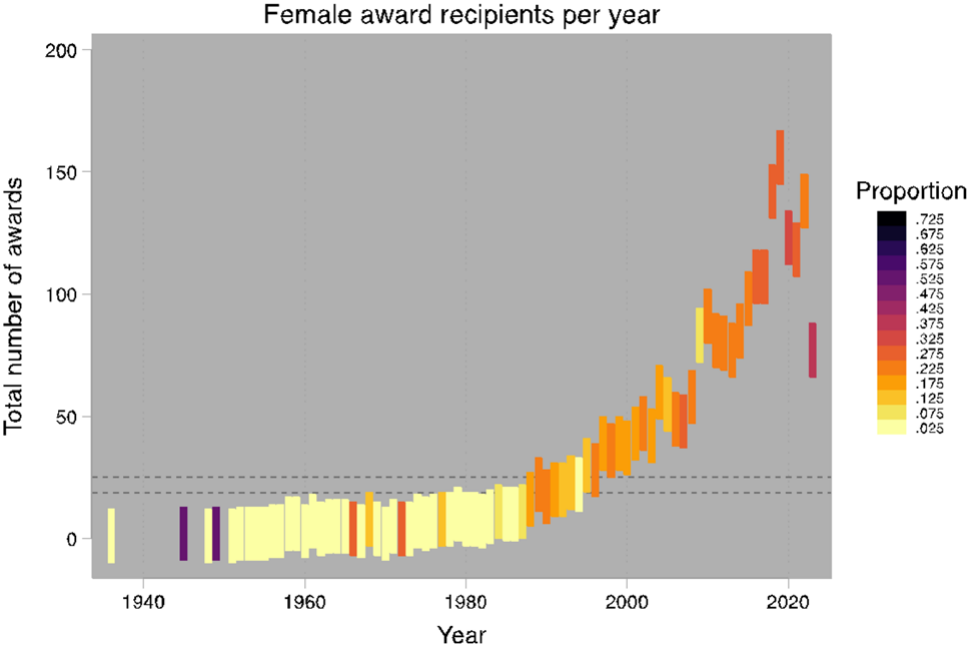
Female award recipients per year

Between 1936 and 2023, there were 2588 awards given out by 22 surgical societies. Of the 2588 awards, 2024 (78.2%) were awarded to male surgeons and 564 (21.8%) were awarded to female surgeons. The percentage of women versus men who won awards for each society is depicted in Fig. 1. The number of women who have received awards per year since 1936 ranges from 0 to 25, with the proportion of female awardees ranging from 0 to 0.5, compared to their male counterparts (Fig. 2). The proportion of awardees who were women has increased significantly since 1936 and continues to increase significantly in the last two decades (*p* < 0.01) (Fig. 3). For every year since 2006, the proportion of women who won awards has increased by 0.7% (95% CI 0.007–0 0.008, *p* < 0.001), when controlling for surgical society.

The odds of a woman receiving an award from a surgical society have increased by 3% every year since 2006 (OR 1.03, 95% CI 1.01–1.07, *p* = 0.004). For example, the proportion of awards that went to female surgeons in 2006 was 0.22, and that proportion increased to 0.35 in 2023. The breakdown of award receipt gender and surgical society is outline in Table 1. As shown below, most recipients are overwhelmingly male, and some societies have no female recipients.

**Table 1:** Society awards by year

Society	Total Awards	Years	Male	Female
AAES	35	1985–2017	24	11
AANS	136	2008–2022	131	5
AOAO	177	2010–2022	169	8
ACS	147	1957–2009	131	16
APSA	118	2013–2023	86	32
AAHS	82	1996–2007	55	27
ASBrS	12	2015–2020	2	10
ASIA	207	1987–2023	150	57
IHPBA	18	2004–2022	18	0
AANA	84	2010–2022	76	8
SSAT	47	1987–2023	38	9
ASMBS	66	2000–2019	60	16
SUS	197	1987–2011	119	78
AHPBA	11	2012–2023	10	1
AAST	239	1999–2012	177	62
EAST	52	2012–2023	30	22
AAS	105	1936–2022	88	17
ASPS	198	1984–2019	185	13
AAOS	21	2017–2023	19	2
AATS	385	1993–2023	277	108
SAGES	250	2008–2022	188	62

## Discussion

Awards plays a crucial role in medicine as they highlight one’s accomplishments, as they not only celebrate a physician’s achievements but also impact career advancement, professional growth, networking opportunities, research funding, and leadership positions. However, the gender gap in surgical societies awards within medicine and surgery is still heavily disproportionate between men and women.

In our analysis, we observed that although a gender gap persists in the allocation of surgical society awards, there has been notable progress in recent decades. Our data indicate a substantial rise in the numbers of female awardees from 1936, with a significant acceleration over the past twenty years. Since 2006, we found a yearly increase of 0.7% in the proportion of awards granted to women, after adjusting for the surgical society variable. Furthermore, the likelihood of a female surgeon receiving an award has risen annually by 3% since 2006.

Past studies over the years have consistently indicated the gender disparities in awards by medical societies. In 2017, Silver et al., study revealed the significance of underrepresentation of female physicians in the recipient of major award recognition awards. In a study spanning 54 journals and 28 medical specialties, researchers found that women’s participation on editorial and professional society boards does not consistently mirror their presence in the respective fields. Moreover, they observed a severe underrepresentation of women in leadership roles within certain specialties, even in fields where they are significantly present, [6].

Another study further illustrated this notion of the underrepresentation of women within social society awards explaining that even when women are recognized, female surgeons still tend to be less represented in the most prestigious categories, including lifetime achievement award with just 12.9% of female surgeons receiving it [7]. The authors examined awards from 20 surgical societies over two decade and found that of 1642 awards given, 1222 (74.3%) were given to men and only 420 (25.6%) were given to women [7]. This broad research on gender disparities included awards from 11 societies in 7 medical specialties by Silver et al., and in Atkinson et al.,’s study of 20 surgical societies, over a 20-year period [7]. In comparison to our research, our results highlight “some” advancement in the recognition of women by surgical societies. Nonetheless, these findings still indicate very slow progress as the available data from 22 surgical societies with an 87-year period (1936 to 2023) shows a considerable gender gap in surgical societies.

Besides being underrepresented in social society awards, women face disparities in high-ranking surgical journals as well. In 2011, Amrein et al., evaluated this disparity further by analyzing the editorial boards of 60 medical and surgical journals. The authors found that out of 4175 editorial boards, only 15.9% of female editors-in-chief were women while 17.5% of all board members were women [8]. In 2021, another study examined the proportion of women in editors-in-chief, associate editors, and editorial board members of 50 American surgical journals for a single time period (2020). Of the 50 journals, 8 were excluded due to missing data, however for the 42 journals that had available data, 14.8% of board members were women and only 4.8% of editor-in-chiefs were women [9, 10].

Finally, Bevilacqua et al. 2022 revealed the shortage of women editor-in-chief and board members between 2010 and 2020 [11]. According to this study, 11% of women hold editors-in-chief positions in surgical journals, and 18.9% when both medicine and OBGYN are considered showing a minor improvement over ten years [11]. It is believed that this underrepresentation has a significant impact on their publications because journal editors are regarded as experts in their fields; therefore, failing to position them in high regard and authority would negatively affect their publications. There are once again disparities demonstrated, as despite women making greater contributions to medicine, there is still a lack of recognition, acknowledgement, and representation as editor-in-chief of senior journals, which may contribute to the gender gap in surgical society awards.

The dominance of males in medical research authorship reflects the historical nature of the general medical profession. Consequently, female surgeons still face disparities in authorship. It is worth noting, however, that there are some studies related to specific surgical areas, such as general surgery, as Hart et al., who observed the female composition of the top 19 general surgery journals over the course of a decade [12]. His studies examined the period from 2008 to 2017 and found that the percentage of female first authors in that period increased from 22.9% in 2008 to 30.8% in 2017 [12, 13]. Despite this apparent increase, there remains a gender gap of less than 10% within a nine-year period.

Likewise, Brown et al., 2019 examined six major orthopedics journals from 1987 to 2017 to determine the gender disparity for women as first or senior authors. It was found in his study that in 2007, female orthopedic surgeons represented only 0.80% (1 out of 125) of senior authors as opposed to 5.2% (1216 out of 23,269) of practicing female surgeons. Comparatively, female orthopedic surgeons were responsible for only 1.2% (2 out of 162) of senior authorships while representing 6.5% (2511 out of 36,124) of practicing surgeons ten years later [14–16]. It is evident from this study that female orthopedic surgeons are still underrepresented as senior authors after a period of over 30 years.

Conclusively, a study by Bernardi et al., 2020, which examined female representation in surgery and female authors in peer-reviewed literature for the period 2000 to 2017, revealed that women in the surgical field lag behind men in authorship despite an increase in females entering the field. A study of 560 manuscripts that were peer-reviewed by 195 journals revealed that 24.8% of the first authors and 16.3% of the last authors were females [16–18].

While this study shows some progress in the gender gap in surgical society awards. This study does have limitations, one being that this study is retrospective study as the data collection was only retrieved from the surgical society websites. Secondly, the lack of transparency on the surgical websites by not showing all individuals that were nominated, instead only focusing award recipients. Thirdly, gender determination was limited to the binary male or female, as there were no identifications of pronouns especially for gender neutral names. Which is not inclusive of all potential identifications.

Our study showed the prevalence of gender gaps in surgical society awards to female surgeons, which justifies a continuous and rigorous future research to understand the impact of the disparities in surgical practice and healthcare delivery. While limited data constrain our current analysis, they nevertheless point to a concerning trend. Future research should prioritize the collection of information on nomination utilized by surgical societies, transparency on surgical websites on the number of nominees and the process used to determine award recipients. This will aid us in understanding the impact of award recognition and the extent to which gender gap in recognition correlates with career satisfaction, professional development, and health care delivery, informing targeted interventions to promote gender equity in surgery.

## Conclusion

The continuous underrepresentation of female surgeons in Surgical Society awards is a crucial issue that needs to be addressed. While there has been some progress in recent years with a gradual increase of female recipients, surgical societies need to be more intentional in their selection process. Closing this gender gap is not just a matter of fairness but essential for the field’s advancement.
